# Positive Association Between Hepatitis C Infection and Oral Cavity Cancer: A Nationwide Population-Based Cohort Study in Taiwan

**DOI:** 10.1371/journal.pone.0048109

**Published:** 2012-10-25

**Authors:** Fu-Hsiung Su, Shih-Ni Chang, Pei-Chun Chen, Fung-Chang Sung, Shiang-Fu Huang, Hung-Yi Chiou, Chien-Tien Su, Cheng-Chieh Lin, Chih-Ching Yeh

**Affiliations:** 1 School of Public Health, College of Public Health and Nutrition, Taipei Medical University, Taipei, Taiwan; 2 Department of Family Medicine, Taipei Medical University Hospital, Taipei, Taiwan; 3 The Ph.D. Program for Cancer Biology and Drug Discovery, China Medical University, Taichung, Taiwan; 4 Institute of Biomedical Sciences, Academia Sinica, Taipei, Taiwan; 5 Management Office for Health Data, China Medical University Hospital, Taichung, Taiwan; 6 Graduate Institute of Epidemiology and Preventive Medicine, National Taiwan University College of Public Health, Taipei, Taiwan; 7 Department of Public Health, China Medical University, Taichung, Taiwan; 8 Department of Otolaryngology, Head and Neck Surgery, Chang Gung Memorial Hospital, Chang Gung University, Tao-Yuan, Taiwan; 9 Center of Excellence for Cancer Research, Taipei Medical University, Taipei, Taiwan; 10 Department of Family Medicine, China Medical University Hospital, Taichung, Taiwan; 11 School of Medicine, College of Medicine, China Medical University, Taichung, Taiwan; 12 Department of Healthcare Administration, College of Health Science, Asia University, Taichung, Taiwan; MOE Key Laboratory of Environment and Health, School of Public Health, Tongji Medical College, Huazhong University of Science and Technology, China

## Abstract

**Objectives:**

The association between viral hepatitis (B and C) and oral cavity cancer has been widely debated. This nationwide, population-based cohort study assessed the subsequent risk of oral cavity cancer among patients with chronic viral hepatitis infection.

**Materials and Methods:**

Data were retrieved from insurance claims data of 1,000,000 randomly sampled individuals covered under the Taiwan National Health Insurance system. We identified a total of 21,199 adults with chronic viral hepatitis infection (12,369 with HBV alone, 5,311 with HCV alone, and 3,519 with HBV/HCV dual infections) from 2000–2005. Comparison group comprised 84,796 sex- and age-matched subjects without viral hepatitis during the same study period. Incidence and risk of subsequent oral cavity cancer were measured until 2008.

**Results:**

The incidence of oral cavity cancers was 2.28-fold higher among patients with HCV alone than non-viral hepatitis group (6.15 versus 2.69 per 10,000 person-years). After adjusting for sociodemographic covariates, HCV alone was significantly associated with an increased risk for oral cavity cancer (hazard ratio (HR) = 1.90, 95% confidence interval (CI) = 1.20–3.02). This positive association was highest among individuals in the 40–49-year age group (HR = 2.57, 95% CI = 1.21–5.46). However, there were no significant associations between HBV alone or HBV/HCV dual infections and risk for oral cavity cancer.

**Conclusion:**

Our data suggest that HCV but not HBV infection is a risk factor for oral cavity cancer. In addition, subjects with HCV infection tend to be at early onset risk for oral cavity cancer. This finding needs to be replicated in further studies.

## Introduction

Oral cavity cancer comprises of 2% to 3% of all malignancies (reviewed in Kademani) [1]. Oral squamous cell carcinoma (OSCC) is the most common type of oral carcinoma and accounts for approximately one ninth of oral malignancies. The identified risk factors of oral cavity cancer include tobacco use, alcohol consumption, race, chewing of betel leaves and areca nuts, and low socioeconomic status (SES). Recently, the role that viruses play in the development of oral cavity cancer has also received tremendous interest [1], [2], [3].

Overall, 12% of the global cancer burden is conservatively estimated to be virus-attributable [4]. Currently, six human viruses have been classified by the International Agency for Research on Cancer (IARC) as being carcinogenic to humans based on sufficient evidence supporting their etiologic association with human cancers, namely Epstein-Barr virus, hepatitis B virus (HBV), several types of human papilloma virus (HPV), human T-cell lymphotropic virus type 1, hepatitis C virus (HCV), and Kaposi’s sarcoma-associated herpesvirus [5].

In addition to the association with hepatocellular carcinoma, HCV infection has been associated with other neoplasms including smoking and alcohol-related cancers, such as cancers of the pancreas, lung, and kidney, and the oropharygeal cancer [6] and non-Hodgkin lymphoma [7]. HBV infection also has been associated with intrahepatic cholangiocaricinoma and non-Hodgkin lymphoma [8], [9] as well as pancreas cancer [10].

Previous studies have provided evidence that HCV infection is associated with the development of oral cavity cancer [11], [12], [13]. In 1995, Nagao et al. demonstrated an increased prevalence of HCV antibody and RNA in OSCC patients [11]. A study conducted at a veterans administration medical center in New Orleans reported that 21.2% of 99 patients with squamous cell carcinoma of the head and neck (SCCHN) were coinfected with HCV, which was significantly higher than previously published data (9.9%) (*P*<0.004) [12]. In another study from Japan that evaluated the prevalence of HCV in 4,402 inpatients requiring oral surgery, Takata et al. found that HCV antibody was more prevalent in patients with oral cavity cancer than in those with impacted teeth before adjustment for age (odds ratio (OR) = 2.433; *P*<0.05); however, this difference was reversed after age adjustment (OR = 0.443; *P*<0.05) [13]. They also suggested that HBV surface antigen was more prevalent in patients with benign oral tumors than in healthy subjects. Thus, there seems to be controversy with respect to the relationship between HCV and HBV infections and oral cavity cancer. Whether there is a potential link between chronic HCV and/or HBV infection and the risk of developing oral cavity cancer, namely OSCC, has yet to be investigated.

In Taiwan, oral cavity cancer ranked as the fifth most prevalent cancer in 2008 and approximately 93% of those patients were men with OSCC [14]. In addition, Taiwan is a region in which HBV infection is endemic and with a high prevalence of HCV infection [15]. Therefore, Taiwan provides a unique setting in which to study the association between hepatitis and oral cavity cancer. In the present study, we used a nationwide population-based insurance dataset to assess the possible association between chronic viral hepatitis infection and oral cavity cancer.

## Materials and Methods

### Data Sources

Data analyzed in this study were retrieved from the Taiwan National Health Insurance Research Database (NHIRD), which is managed by the Taiwan National Health Research Institute (NHRI). In 1995, Taiwan commenced its state-run National Health Insurance (NHI) program [16]. By the end of 1996, this insurance program covered approximately 96% of the total population, and contracted with 97% of the hospitals and 90% of the clinics on the island [17]. The Taiwan NHIRD comprises claims data of 1,000,000 individuals randomly selected from all insured enrollees. This sample represents the original medical claims for all islanders under the NHI program. With approval from the NHRI, we used data for ambulatory care claims, all inpatient claims, and updated registries for beneficiaries from the year 2000 to the year 2005 in this study. Diagnoses were coded according to the *International Classification of Disease, Ninth revision, clinical Modification* (ICD-9-CM). The database used in this study can be interlinked by the scrambled unique individual’s personal identification number (PIN). The NHRI safeguards the privacy and confidentiality of all beneficiaries and transfers the health insurance data to health researchers after ethical approval has been obtained. In this analysis, access of the NHIRD has been approved by the NHRI Ethics Review Committee.

### Study Sample

**Figure 1:**
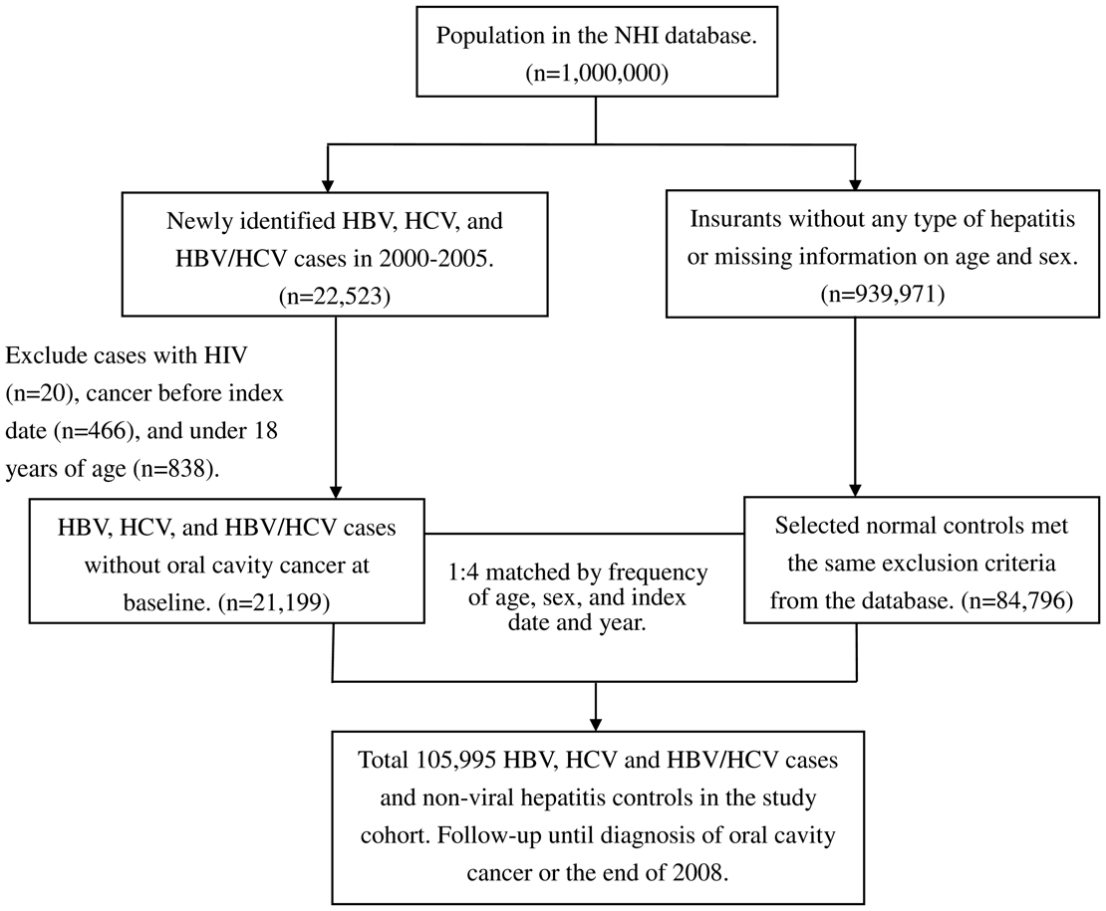
Study flow chart showing the selection of subjects. Footnotes: NHI, National Health Insurance.

Our data consisted of NHIRD data during the period 1996 to 2008. In order to limit our study sample to the adult population, we only selected patients older than 18 years of age. In this study, we identified patients with newly identified HBV (ICD-9-CM: 070.2, 070.3, V02.61) and HCV (ICD-9-CM: 070.41, 070.44, 070.51, 070.54, V02.62) infections during the period of 2000–2005 as the exposure group. The index date for the patients with HBV and HCV was the date of their first medical visit. We excluded subjects with a diagnosis of HIV (ICD-9-CM: 042, 043, 044, V08 and 795.8). Subjects with a diagnosis of malignant neoplasm (ICD-9-CM: 140–230) that had been established before the index date and subjects with missing information on age or sex were excluded. After excluding 20 cases of HIV, 466 cases of cancer diagnosed before index date, and 838 subjects under 18 years of age, 12,369, 5,311, and 3,519 subjects with HBV, HCV and HBV/HCV dual infections were enrolled in this study. For the comparison group, we used a systematic random sampling method to select 4 insured people without viral hepatitis for every insured person with viral hepatitis during the same period. Patients and comparison subjects were age- and sex-matched. To avoid the comparison group being mixed up with new-identified HBV and HCV groups, patients in whom HBV and HCV identified during the follow-up period were excluded. Originally, 939,971 subjects were retrieved from the NHIRD after excluding any type of chronic hepatitis (including diagnoses of HBV and HCV and chronic hepatitis (ICD-9-CM: 070.9, 571.4, 571.8, and 571.9)) or missing information on age and sex. A total of 84,796 non-viral hepatitis subjects were finally enrolled in this study with same exclusion criteria of the exposure group (Fig. 1).

### Statistical Analysis

We first compared the distribution of sociodemographic factors (sex, age, occupation, monthly income, and the geographical location, and the urbanization level of the community in which the patient resided) and the proportions of comorbidities between the cohorts with and those without viral hepatitis (HBV and HCV). Potential baseline comorbidities, including diabetes mellitus (DM) (ICD-9-CM: 250), coronary artery disease (CAD) (ICD-9-CM: 410–414), hyperlipidemia (ICD-9-CM: 272), and hypertension (ICD-9-CM: 401–405), were also ascertained in patients with and without chronic viral hepatitis by searching for the presence of diagnostic codes at any time starting in fiscal year 2000.

The incidence rates of oral cavity cancer in the three groups of patients with viral hepatitis (HBV alone, HCV alone, and HBV/HCV dual infections) were calculated in the follow-up period until the end of 2008. Follow-up time (in person-years) was calculated for each person until oral cavity cancer was diagnosed or censored for death, migration, or discontinued enrolment in the NHI database. The rate ratio was determined under the Poisson assumption. Crude and adjusted hazard ratio (HR) and 95% confidence interval (CI) for factors associated with risk of oral cavity cancer were estimated using univariate and multivariate Cox proportion hazard regression models. To address the hypothesis of the presence of an association between viral hepatitis and oral cavity cancer, interaction between viral hepatitis status and sex as well as the age of developing oral cavity cancer was tested in the Cox models. All analyses were performed by SAS statistical software for Windows (Version 9.1; SAS Institute, Inc., Cary, NC, USA), and the significance level was set to be 0.05.

## Results

### Baseline Characteristics and Comorbidities of the Study Subjects

In our study, the patients with HCV infection were more likely to be older, blue collar workers, living in lower urbanization level areas in southern Taiwan, as well as to have lower income levels than the 84,769 non-viral hepatitis comparison subjects (Table 1). They also were more likely to have DM, hypertension, and hyperlipidemia. On the contrary, patients with HBV tended to be younger and to have a lower prevalence of CAD and hypertension than subjects in the non-viral hepatitis comparison group.

**Table 1:** Baseline characteristics and comorbidities between viral hepatitis and non-viral hepatitis adult groups identified in 2000–2005.

	Viral hepatitis								
	No		HBV alone		HCV alone		HBV+HCV		
	N = 84796		N = 12369		N = 5311		N = 3519		
Variables	n	(%)	n	(%)	n	(%)	n	(%)	p-value†
Sex									<0.0001
Women	37444	(44.2)	5145	(41.6)	2653	(50.0)	1563	(44.4)	
Men	47352	(55.8)	7224	(58.4)	2658	(50.1)	1956	(55.6)	
Age, years									<0.0001
18–29	18200	(21.5)	3472	(28.1)	527	(9.9)	551	(15.7)	
30–39	17556	(20.7)	3113	(25.2)	668	(12.6)	608	(17.3)	
40–49	19068	(22.5)	2842	(23.0)	1120	(21.1)	805	(22.9)	
50–59	13760	(16.2)	1619	(13.1)	1113	(21.0)	708	(20.1)	
60–69	10316	(12.2)	883	(7.1)	1105	(20.8)	591	(16.8)	
70–79	5032	(5.9)	380	(3.1)	660	(12.4)	218	(6.2)	
≥80	864	(1.0)	60	(0.5)	118	(2.2)	38	(1.1)	
Geographic region									<0.0001
Northern	40020	(47.2)	5468	(44.2)	1540	(29.0)	1126	(32.0)	
Central	16791	(19.8)	2672	(21.6)	1153	(21.7)	729	(20.7)	
Southern	21349	(25.2)	3297	(26.7)	2196	(41.4)	1401	(39.8)	
Eastern and Islands	6635	(7.8)	932	(7.5)	422	(8.0)	263	(7.5)	
Occupation									<0.0001
Public	8132	(9.6)	1490	(12.1)	419	(7.9)	339	(9.6)	
Labor	27421	(32.3)	3671	(29.7)	2522	(47.5)	1488	(42.3)	
Business	37891	(44.7)	5802	(46.9)	1611	(30.3)	1268	(36.0)	
Low income	371	(0.4)	46	(0.4)	49	(0.9)	19	(0.5)	
Retired and others	10981	(13.0)	1360	(11.0)	710	(13.4)	405	(11.5)	
Urbanization level									<0.0001
1	25896	(30.5)	3627	(29.3)	1069	(20.1)	804	(22.9)	
2	24648	(29.1)	3712	(30.0)	1530	(28.8)	1001	(28.5)	
3	15623	(18.4)	2327	(18.8)	888	(16.7)	625	(17.8)	
4	18621	(22.0)	2702	(21.9)	1823	(34.3)	1089	(31.0)	
Monthly income, NT$									<0.0001
0	18817	(22.2)	2560	(20.7)	1147	(21.6)	722	(20.5)	
1–15840	10378	(12.2)	1280	(10.4)	637	(12.0)	353	(10.0)	
15841–25000	36597	(43.2)	5043	(40.8)	2743	(51.7)	1752	(49.8)	
>25000	19004	(22.4)	3486	(28.2)	784	(14.8)	692	(19.7)	
Diabetes mellitus									<0.0001
No	78312	(92.4)	11364	(91.9)	4320	(81.3)	2991	(85.0)	
Yes	6484	(7.7)	1005	(8.1)	991	(18.7)	528	(15.0)	
Coronary artery disease									<0.0001
No	37983	(44.8)	6495	(52.5)	2468	(46.5)	1757	(49.9)	
Yes	46813	(55.2)	5874	(47.5)	2843	(53.5)	1762	(50.1)	
Hypertension									<0.0001
No	69159	(81.6)	10479	(84.7)	3463	(65.2)	2575	(73.2)	
Yes	15637	(18.4)	1890	(15.3)	1848	(34.8)	944	(26.8)	
Hyperlipidemia									<0.0001
No	76023	(89.7)	10797	(87.3)	4289	(80.8)	2905	(82.6)	
Yes	8773	(10.4)	1572	(12.7)	1022	(19.2)	614	(17.5)	

Urbanization level: 1 indicate the highest level of urbanization and 4 the lowest.

† Chi-square test.

### Incidence Densities and HR of Oral Cancer among Viral Hepatitis

In total, we observed 198 cases of oral cavity cancer (174 males and 24 females) among 682,647 person-years, with an incidence density of 2.90 per 10,000 person-years (Table 2). The univariate Cox proportional hazard regression analysis revealed that the risk of developing oral cavity cancer among men with hepatitis was more than 5-fold higher than among women with hepatitis (HR = 5.88, 95% CI = 3.84–9.01). The highest age-specific HR was observed in the 50–59-year age group (HR = 15.9, 95% CI = 6.36–39.5). People residing in eastern Taiwan and those residing on offshore islands had 2.99-fold higher risk (95% CI = 1.88–4.75) of developing oral cavity cancer than individuals living in northern Taiwan. Blue collar workers tended to have a significantly higher risk of developing oral cavity cancer than white collar workers (HR = 1.65, 95% CI = 1.20–2.27). Subjects with a monthly income ranging from 1–15841 NT dollars were at the highest of developing oral cavity cancer (HR = 2.49, 95% CI = 1.45–4.27) among the income groups. The incidence of oral cavity cancer was approximately 2.3-fold higher in the cohort of patients with HCV infection than subjects in the non-viral hepatitis group (6.15 versus 2.69 per 10,000 person-years) (HR = 2.28, 95% CI = 1.44–3.60). There was no significant association between patients with HBV alone or patients with HBV+HCV dual infections and risk of developing oral cavity cancer. In addition, baseline comorbidities, including DM, CAD, hyperlipidemia, and hypertension were not significant risk factors for developing oral cavity cancer.

**Table 2:** Incidence density of oral cavity cancer for viral hepatitis and non-viral hepatitis adult cohorts identified in 2000–2005.

				Univariate		Multivariate	
	Cases	PY	I^a^	HR	(95% CI)	HR	(95% CI)
Sex							
Women	24	305111	0.79	1.00	(reference)	1.00	(reference)
Men	174	377536	4.61	5.88	(3.84–9.01)***	6.70	(4.34–10.3)***
Age, years							
18–29	5	150172	0.33	1.00	(reference)	1.00	(reference)
30–39	33	143684	2.30	6.91	(2.70–17.7)***	7.43	(2.88–19.2)***
40–49	65	155956	4.17	12.5	(5.05–31.2)***	13.7	(5.47–34.5)***
50–59	58	110782	5.24	15.9	(6.36–39.5)***	17.9	(7.11–45.0)***
60–69	28	81639	3.43	10.4	(4.01–26.9)***	10.6	(4.08–27.6)***
70–79	8	35840	2.23	6.92	(2.26–21.1)**	6.25	(2.03–19.2)***
≥80	1	4572	2.19	7.24	(0.85–62.0)	6.59	(0.77–56.6)
Geographic region							
Northern	53	309978	1.71	1.00	(reference)	1.00	(reference)
Central	45	137697	3.27	1.91	(1.28–2.84)**	1.68	(1.12–2.51)*
Southern	73	182033	4.01	2.34	(1.64–3.34)***	1.98	(1.38–2.84)**
Eastern and island	27	52932	5.10	2.99	(1.88–4.75)***	2.52	(1.58–4.03)***
Occupation							
Public	17	67960	2.50	1.10	(0.65–1.87)		
Labor	85	226777	3.75	1.65	(1.20–2.27)**		
Business	68	299509	2.27	1.00	(reference)		
Low income	1	2916	3.43	1.53	(0.21–11.0)		
Retired and others	27	85484	3.16	1.39	(0.89–2.18)		
Monthly income, NT$							
0	23	146869	1.57	1.00	(reference)	1.00	(reference)
1–15840	31	79023	3.92	2.49	(1.45–4.27)**	1.37	(0.79–2.37)
15841–25000	107	298236	3.59	2.27	(1.45–3.56)**	1.13	(0.71–1.79)
>25000	37	158519	2.33	1.47	(0.88–2.48)	0.65	(0.38–1.12)
Viral hepatitis							
No	147	546846	2.69	1.00	(reference)	1.00	(reference)
HBV alone	21	78803	2.66	1.00	(0.63–1.58)	1.11	(0.70–1.75)
HCV alone	21	34150	6.15	2.28	(1.44–3.60)**	1.90	(1.20–3.02)**
HBV+HCV	9	22848	3.94	1.46	(0.75–2.86)	1.21	(0.62–2.38)

PY, person-years; I, incidence; HR, hazard ratio; CI, confidence interval.

a: incidence rate (per 10,000 person-years).

* *P*<0.05, ** *P*<0.01, *** *P*<0.0001.

Significant predictors in the univariate analysis were included in a multivariate Cox hazard model to identify the most important risk factors for oral cavity cancer (Table 2). Because occupation and monthly income were highly correlated, we selected monthly income for multivariate analysis. In the multivariate Cox hazard model, we found that men had a 6.7-fold greater risk of developing oral cavity cancer than women (HR = 6.70, 95% CI = 4.34–10.3). The highest age-specific HR still remained in the 50–59-year age group (HR = 17.9, 95% CI = 7.11–45.0). Individuals living in eastern Taiwan and islands had significantly highest risk of developing oral cavity cancer (HR = 2.52, 95% CI = 1.58–4.03), whereas monthly income levels were not related to the disease risk. The multivariate Cox model also showed that the risk of developing oral cavity cancer was significantly higher among subjects with HCV infection than among individuals with no hepatitis group (HR = 1.90, 95% CI = 1.20–3.02). Further data analysis showed that HCV was a significant risk factor for oral cavity cancer even after excluding individuals with diagnoses of smoking-related cancers (including ICD-9-CM: 146–150, 157, 160–162, and 189) during the follow-up period (HR = 1.92, 95% CI = 1.21–3.04) (not shown in the tables).

### Adjusted HRs of Oral Cavity Cancer with Viral Hepatitis by Age and Sex Stratification

Table 3 shows the age-specific and sex-specific HRs of oral cavity cancer for hepatitis cohorts compared to the comparison cohort. The adjusted HRs of oral cavity cancer among subjects infected with HCV alone were highest in those aged 40–49 years (HR = 2.57, 95% CI = 1.21–5.46) followed by those <40 years old (HR = 2.48, 95% CI = 0.75–8.19). However, no significant associations appeared for those with HBV alone or those with HCV/HBV dual infections. Furthermore, the sex-specific adjusted HRs of oral cavity cancer associated with HCV were 1.69 (95% CI = 0.49–5.83) for women and 1.88 (95% CI = 1.14–3.09) for men. On the contrary, there were no significant risks of oral cavity cancer in those with sole HBV infection or those with HBV/HCV dual infections. The interaction between HCV status and age of developing oral cavity cancer was moderately significant (*P* = 0.078). In the contrast, the interaction between HCV status and sex was not statistically significant (*P* = 0.97).

**Table 3:** Adjusted HRs and 95% CIs of oral cavity cancer associated with viral hepatitis compared with non-viral hepatitis by age and sex stratification.

	Viral hepatitis					
	HBV alone		HCV alone		HBV + HCV	
	aHR	(95% CI)	aHR	(95% CI)	aHR	(95% CI)
Age, years^a^						
<40	1.01	(0.39–2.63)	2.48	(0.75–8.19)	1.86	(0.44–7.84)
40–49	0.85	(0.36–1.98)	2.57	(1.21–5.46)*	0.93	(0.22–3.82)
50–59	1.24	(0.53–2.92)	1.67	(0.70–3.95)	1.55	(0.55–4.34)
≥60	1.86	(0.65–5.33)	1.20	(0.42–3.45)	0.65	(0.09–4.76)
Interaction		p = 0.16		p = 0.078		p = 0.61
Sex^b^						
Women	1.18	(0.27–5.12)	1.69	(0.49–5.83)	1.18	(0.16–8.92)
Men	1.08	(0.66–1.75)	1.88	(1.14–3.09)*	1.28	(0.63–2.62)
Interaction		p = 0.83		p = 0.97		p = 0.90

aHR: adjusted hazard ratio.

a: adjusted for sex, region, and income.

b: adjusted for age, region, and income.

* *P*<0.05, ** *P*<0.01.

## Discussion

In this nationwide, population-based cohort study conducted in an area where both viral hepatitis and oral cavity cancer are endemic, significant risk of developing oral cavity cancer was observed in patients with HCV but not HBV infection. We also found that, in the general Taiwanese population, male subjects tended to have a 6.7-fold higher risk of developing oral cavity cancer than female counterparts. This finding is compatible with the finding reported in the Taiwan government cancer registry report [14]. The age of developing oral cavity cancer among HCV carriers (peak age group: 40–49 years) also tended to be younger than that of the general population (peak age group: 50–59 years). We also observed that subjects residing in the eastern Taiwan and islands had significantly highest risk of developing oral cavity cancer, followed by those living in the southern part of Taiwan.

HCV infection predisposes patients to extrahepatic disorders involving renal, dermatologic, hematologic, and rheumatologic systems as well as autoimmune abnormalities [18], [19]. Extrahepatic manifestations may result from immunologic trigger mechanisms as well as viral invasion and replication that affect extrahepatic tissues and organs [20].

The association between HCV infection and oral cavity cancer is still controversial [11], [12], [13]. HCV is a virus with triple tissue tropism – hepatotropism, lymphotropism, and sialotropism [21]. HCV RNA has been detected in saliva and salivary tissue in patients with chronic salivary gland disorders, a finding that suggests that HCV, a sialotropic virus, can reside within salivary gland cells [22]. Other studies, however, have shown that HCV detection in saliva and in salivary glands is not related to oral health conditions [23], [24], [25]. Grossmann et al. suggest that HCV might play an indirect role in causing diseases of the oral cavity and salivary glands by stimulating an immune response [21]. In addition, one of the most frequently reported oral extrahepatic manifestations of HCV infection is lichen planus [26], [27], [28]. This premalignant condition is associated with the development of OSCC [29]. In a Brazilian cross-sectional study, there was a significant association between oral lichen planus and hepatitis C in their cohort of 215 patients with chronic HCV infection [21]. The progression of lichen planus into OSCC in patients with hepatitis C has been described in two case reports [29], [30]. Several studies have shown that the transformation rate of oral lichen planus to OSCC is approximately 0.04% to 1.74% [31], [32]. Interestingly, Nobles et al. demonstrated no predilection for the development of oral cavity cancers in patients infected with HCV [12]. Therefore, the role that HCV and its viral proteins play in the pathogenesis of oral diseases remains unclear.

Furthermore, Nagao et al. reported a higher prevalence of HCV antibody and RNA in patients with oral cavity cancer [11]. However, in 2002, Takata et al. reported, after age adjustment, a significantly decreased prevalence of HCV antibody in their patients with oral cavity cancer [13]. Using logistic regression analysis to eliminate the influence of age, Takata and colleagues suggested that the increased prevalence of HCV antibody in oral cavity cancer patients is more likely due to age differences rather than to carcinogenic action of HCV [11], [27], [28]. In addition, Nobles et al. observed that HCV patients present with an earlier age of onset of squamous cell carcinoma of the head and neck (SCCHN) than controls [12]. The age difference between these two groups may reflect differences in risk factors for the development of hepatitis C. The increased prevalence of intravenous drug abuse among younger populations, and socioeconomic differences between young and old age groups may also explain the age difference. Nobles and his colleagues also suggested HCV is considered a cofactor instead of a comorbid condition in relation to the development of head and neck cancer [12]. The localization of HCV RNA in oral lichen planus and OSCC tissue derived from patients with hepatitis C may provide one possible explanation to oral carcinogenesis of HCV [28]. In this prospective cohort study, we demonstrated that HCV is a risk factor for the development of oral cavity cancer (HR = 2.57, 95% CI = 1.21–5.46 in the 40–49–year age group). The risk of developing oral cavity caner among patients with HCV is 10 years earlier than the general population. This finding is unlike the finding reported in the retrospective case study by Takata and his colleagues [13]. In addition, multivariate Cox regression analysis revealed that males had a 6.7-fold greater risk of developing oral cavity cancer than females. The discrepancy between men and women in the general population can be explained by the fact that men tend to be engaged in riskier lifestyle behaviors than women, such as cigarette smoking (46.8% vs. 4.3%), frequent alcohol consumption (15.1% vs. 2.6%), and betel quid chewing (14.4% vs. 1.5%) [33], [34], [35]. However, among the HCV infected subjects, female carriers are at similar risk of developing oral cavity cancer to their male counterparts (HRs were 1.69 and 1.88, respectively). Cacoub et al. suggested that female sex is one of the risk factors associated with developing extrahepatic manifestations of HCV infection [36]. Although another possibility to explain the stronger association of HCV with oral cancer for females compared to males might be due to the small number of female oral cancer subjects in our cohort, this finding provided an interesting observation. Further studies with larger female samples are needed to verify this observation.

In this study, infection with HBV alone and HBV/HCV coinfection were not correlated with oral cavity cancer. Our finding is consistent with that reported in a Japanese study, which showed that high levels of HBV surface antigen were observed in patients with benign oral tumors but not in patients with oral cavity cancer requiring dental surgery [13]. Their finding suggests that HBV infection is unlikely to play a major role in oral tumor formation. A study by Bokor-Bratic also suggested that oral leukoplakia was not associated with HBV infection in Serbia [37]. In addition, only HCV has the lymphatrophic character that is assumed to be the cause of HCV-associated extrahepatic manifestations [38]. Therefore, this HCV’s lymphatrophic character may explain why we found that HCV, but not HBV, was associated with oral cavity cancer. HBV and HCV are both hepatrophic viruses. Their coinfection is associated with clinically and histologically more severe liver disease and higher risk for the development of hepatocellular carcinoma [15], [39]. Cho et al. have recently demonstrated in a meta-analysis study that co-infection of HBV and HCV has a subadditive risk for HCC [40]. Other clinical studies suggested a reciprocal interference of one virus on the replication of the other or both [41], [42]. Some other clinical studies also showed co-infected cases with the phenomenon of HBV dominant or HCV dominant effect [43], [44]. This reciprocal interference between HBV and HCV may also explain the lack of increasing incidence of oral cavity cancer among our HBV+HCV infected population as one or both viruses were inhibited for their replication. Further prospective cohort studies are required to verify this finding.

This study has 2 main strengths. First, our study is the very first one used complete nationwide population-based data to assess the association between chronic viral hepatitis infection and oral cavity cancer. The sample size is large to differentiate risk difference between those with HBV and HCV infections. Second, selection and nonresponse biases may have been minimized by the comprehensive coverage of the NHI system (>96% of the islanders) and the large sample size.

This study had several limitations. First, some patients with hepatitis infection do not have obvious clinical symptoms and, therefore, might not seek medical attention. Claims for medical services would, therefore, not be available for those patients. As a result, some patients with asymptomatic hepatitis infection were most likely included in the comparison group. However, if viral hepatitis infection is associated causally with oral cavity cancer, this misclassification may lead the estimated HRs toward the null and further strengthen our findings. In addition, the overall seroprevalence of antibody to HCV (anti-HCV) in Taiwan has been estimated approximately <3% [45], [46]. The prevalence of chronic HCV infection identified in our cohort was approximately 2.2%. Therefore, the positive association between HCV and oral cavity cancer in our cohort is accountable. Second, some oral cavity cancer risk factors, such as chewing of betel nut, smoking, and alcohol consumption were unavailable in the insurance claims database. Therefore, we cannot rule out some of the potential confounding effects associated with these factors. Ko et al. suggested that betel quid chewing is the most potent risk factor for oral cavity cancer in Taiwan, followed by cigarette smoking and alcohol drinking [47]. In Taiwan, the prevalence of cigarette smoking and betel quid chewing is highest in the eastern region, followed by the southern region [33]. Betel nut chewers in Taiwan are who tend to be poorly educated, low income earners, and blue collar workers [33]. We, hence, included monthly income and geographical regions in our multivariate Cox hazard model to reduce confounding effects caused by betel quid chewing, cigarette smoking, and alcohol consumption. In addition, in Taiwan, almost all betel quid chewers are smokers [33]. After excluding smoking-related cancers among our subjects, we found that the HR of oral cavity cancer for HCV alone infection increased from 1.90 to 1.92. Therefore, these lifestyle factors may not significantly confound our results as we thought. Third, the diagnoses of oral cavity cancer, HBV infection, HCV infection, and other comorbidities based on International Classification of Disease codes may be less accurate than those obtained through a standardized procedure. However, the NHI Bureau of Taiwan randomly samples a fixed percentage of claims from every hospital and randomly interviews patients and reviews charts each year to verify the diagnosis validity and quality of care [48]. Patients with confirmed oral cavity cancer deserve medical cares as the “Catastrophic Illness” with minimum co-payment under the Taiwan NHI plan. All cancers are histology confirmed. The diagnoses of oral cavity cancer are likely accurate and are representative of all oral cavity cancer in Taiwan. Fourth, the vast majority of the residents in Taiwan are of Chinese ethnicity. Hence, the ability to generalize the results to other racial/ethnic groups is unclear given that the transmission route of viral hepatitis infection in Chinese might not be the same as that in other ethnic groups. Fifth, there is some plausibility for the association of lichen planus (one of the most frequently reported oral extrahepatic manifestations of HCV infection) and oral cavity cancer [29]. However, the incidence of lichen planus in our subjects was not indexed on the insurance records.

In summary, in this nationwide population-based cohort study conducted in a country in which both viral hepatitis and oral cavity cancer are endemic, we found a positive association between oral cavity cancer and HCV infection. Male subjects were at higher risk of developing oral cavity cancer than their female counterparts. Patients with HCV presented at an earlier age of onset of oral cavity cancer than subjects in the viral hepatitis-free control group. More perspective cohort studies are needed to determine the association between oral cavity cancer and HCV infection.
